# Towards recommendations for arts and health evaluation: a two-part review of Quality of Life scales used in mental health and arts research

**DOI:** 10.3389/fpubh.2026.1933623

**Published:** 2026-10-12

**Authors:** Abielle Shao Hua Liu, Raeanne Chien Yin Wong, Renick Daryl Liebin Lee, Jean C. J. Liu, Nisha Sajnani, Kat R. Agres

**Affiliations:** 1 Centre for Music and Health, Yong Siew Toh Conservatory of Music, National University of Singapore, Singapore, Singapore; 2 Alice Lee Centre for Nursing Studies, Yong Loo Lin School of Medicine, National University of Singapore, Singapore, Singapore; 3 Yong Loo Lin School of Medicine, National University of Singapore, Singapore, Singapore; 4 Health and Social Sciences Cluster, Singapore Institute of Technology, Singapore, Singapore; 5 Duke-NUS Graduate Medical School, National University of Singapore, Singapore, Singapore; 6 Jameel Arts & Health Lab, New York University, New York, NY, United States; 7 Yeo Boon Khim Mind Science Centre, National University of Singapore, Singapore, Singapore

**Keywords:** arts and health, frequency of scales, mental health, Quality of Life, validated scale recommendations

## Abstract

There is increasing integration of the arts in public health, particularly in relation to Quality of Life (QoL). In health research and practice, QoL provides a broad snapshot of how an individual is faring - their subjective perception of how their life is in relation to their goals, expectations, standards, and concerns. To support more rigorous evaluation of arts and health interventions, this two-part review assesses the most widely used QoL scales, provides recommendations on scale adoption, and examines their frequency of use across arts domains. The first part of our review systematically identifies the most widely used QoL scales for the general adult population across the psychiatry, psychology, and mental health literature. According to our literature review, the top four scales for assessing QoL are: Euro-QoL (EQ-5D-3L/5L), SF-36, WHOQOL-BREF, and SF-12, with EQ-5D most frequently used in the target literature. The second part examines the scales’ frequency across 18 art domains, drawn from a broader set of arts domains from the literature. SF-36 is the most commonly used QoL scale for evaluating arts interventions, followed by WHOQOL-BREF, SF-12, and EQ-5D. Music accounts for the largest number of publications involving a QoL scale, while literary festivals and electronic arts interventions yielded the fewest publications.

## 1 Introduction

In recent decades, the integration of arts within public health has gained recognition, particularly in health promotion and health prevention (1). This is supported by a growing body of research demonstrating the health-promoting effects of arts engagement. A 2019 scoping review from the World Health Organization synthesised this evidence, detailing how the arts can help prevent illness, promote health, and support the management and treatment of diverse health conditions (2). Within this context, Quality of Life (QoL) may serve as a useful indicator for assessing the impact of arts interventions on health outcomes as an important determinant of an individual’s wellbeing.

QoL is a subjective evaluation of one’s perception of their life relative to their goals, expectations, standards, and concerns (3). It reflects the interplay of various facets of life, including personal health, relationships, education status, work environment, social status, wealth, sense of security and safety, freedom, autonomy in decision-making, social belonging, and physical surroundings (4). Accordingly, many researchers and clinicians have divided the concept into constituent domains for more accurate and nuanced evaluation, while others have divided QoL into personal wellbeing and life satisfaction (5).

While many validated tools exist for evaluating QoL, their application in the field of arts and health remains inconsistent. Many studies rely on qualitative rather than quantitative data (6), contributing to an inconsistent use of psychometrically sound evaluation tools (7). Moreover, the use of diverse methods, variability in scale choice across studies (8), and the use of bespoke (researcher-developed) scales have resulted in limited guidance for artist-researchers to select appropriate and reliable scales among the plethora of scales available. As interest in the health impacts of arts interventions continues to grow, addressing this methodological gap is essential to support their broader public-health relevance. In 2012, Clift called for the field to move beyond practice-based arts interventions toward building robust evidence bases with precise, measurable outcomes (9). A decade later, a scoping review by Pesata et al. found that this need persists, identifying heterogeneity and lack of standardized outcome measures as key barriers to evidence synthesis and wider public health acceptance (10).

Therefore, in this article, we present a review designed to support more rigorous, consistent evaluation practices. It forms part of a broader toolkit for outcome evaluation in arts and health (11). Specifically, our review seeks to address existing gaps by identifying the most widely used scales for measuring QoL.

## 2 Methods

We carried out a two-part review: (1) sifting through existing review papers to identify the most frequently used QoL scales within the mental health and psychological sciences, and (2) counting the use of each shortlisted QoL scale across different domains of arts and health research. We then examined the key features of each scale to provide recommendations for future research. Our review was conducted and reported in accordance with the PRISMA guidelines (12), where applicable. The overview of our two-part methodology is summarised in Figures 1A, B.

**Figure 1:**
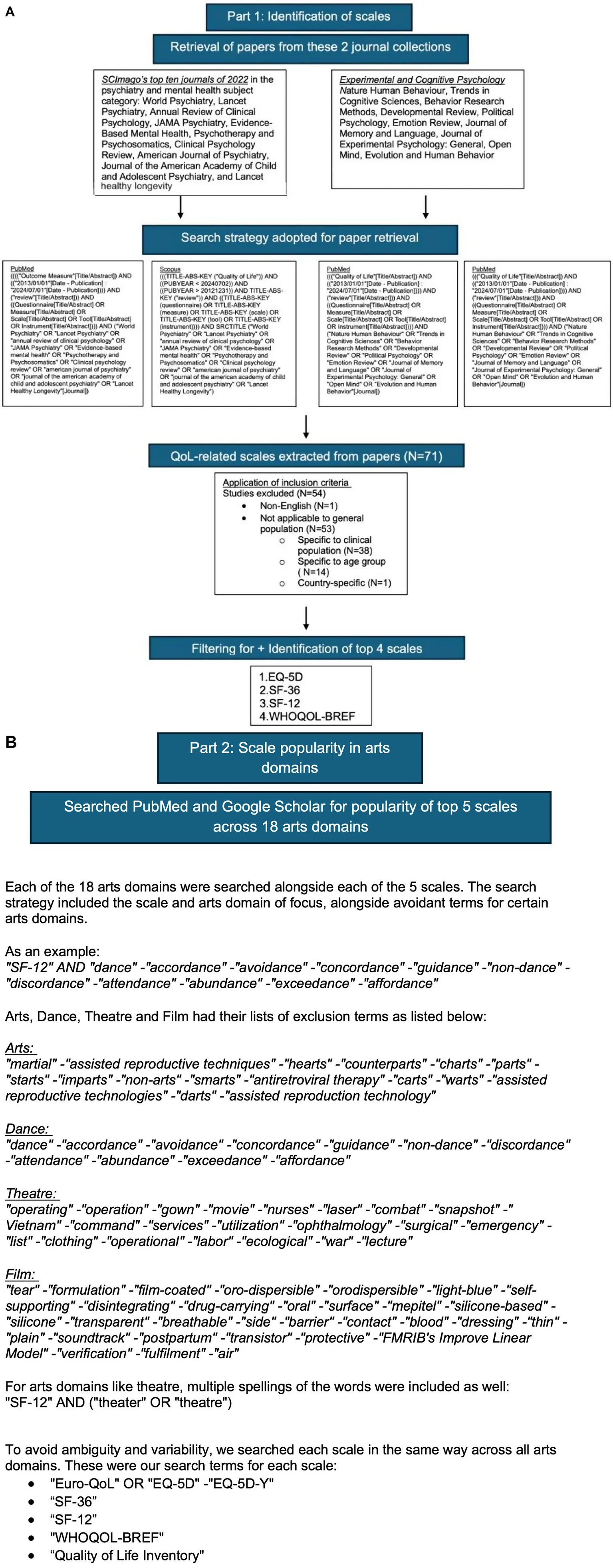
**(A)** Flowchart of methods—Part 1. **(B)** Flowchart of methods—Part 2.

### 2.1 Search of frequently used measures

In part one, we sought to identify the most frequently used measures for assessing Quality of Life within the published literature in mental health and psychological sciences. Extending the method employed in Ilya et al. (13), our search retrieved articles from *SCImago’s* top 10 journals in the Psychiatry and Mental Health category. For this paper, the top 10 journals in Experimental and Cognitive Psychology were intentionally added for an increased focus on Quality of Life (The complete set of journals is listed in Figure 1).

Using both sets of journals provided breadth and depth to our research, allowing us to capture a comprehensive range of scales across multidisciplinary studies. Following Ilya et al. we then documented the frequency with which each measurement tool was used.

Review papers published from 1 January 2013 to 1 July 2024 were retrieved for this literature review, allowing targeted focus on articles most relevant to the search, and to ascertain which tools are currently being used according to the recent literature. The selection of the review papers is illustrated in the PRISMA diagram for the outcomes of QoL in Figure 2.

**Figure 2:**
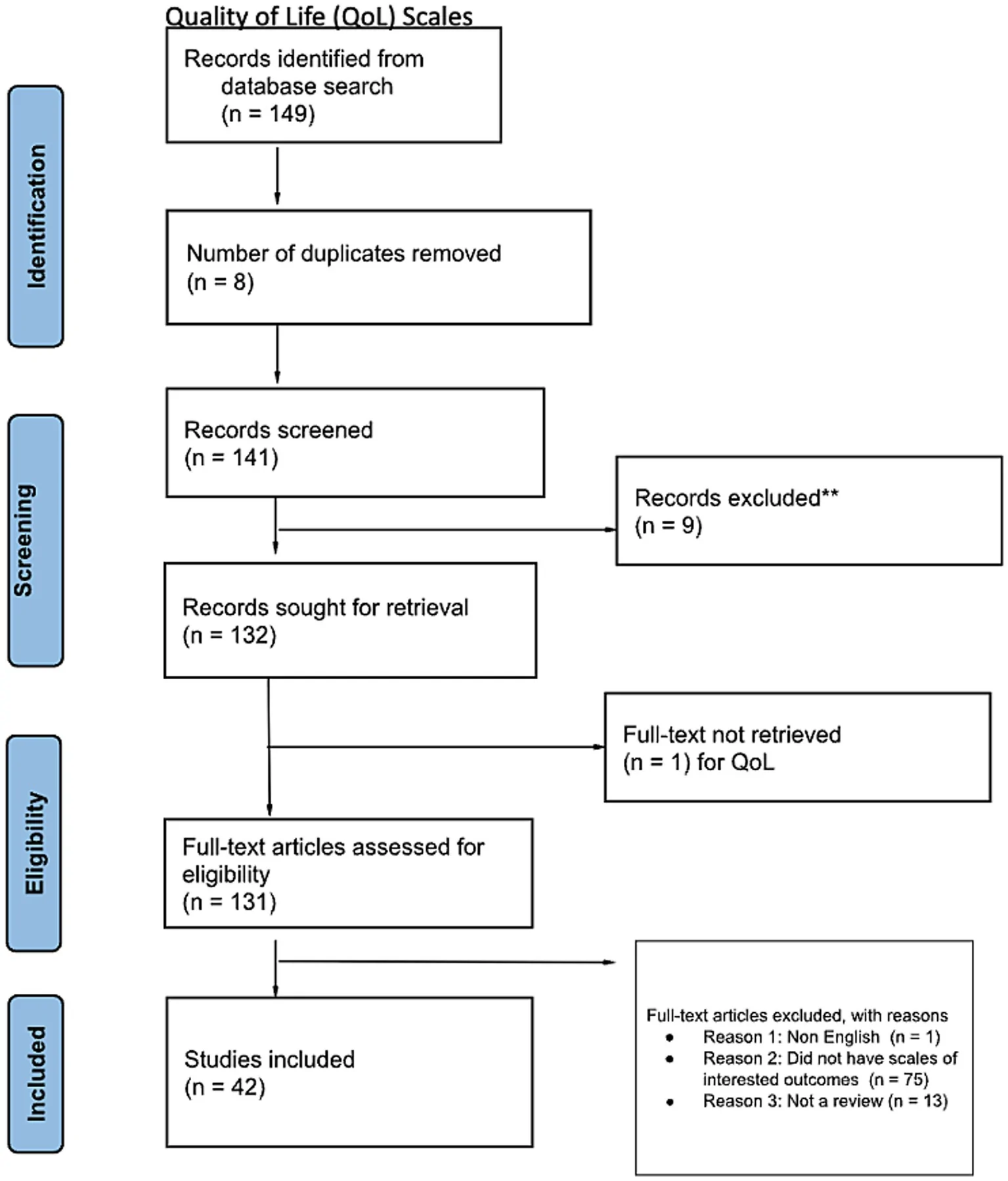
PRISMA Diagram Quality of Life.

In the selection of scales:

Scales from each of the review papers were considered and extracted. They were then evaluated based on four inclusion criteria:

1. Scales had to be mentioned more than twice in the reviews retrieved.
2. Scales had to be available in English and widely administered in this language.
3. Scales must be applicable to the general population. This would exclude scales for specific clinical populations, specific age groups, and scales not validated internationally.
4. Scales do not require clinicians or medically trained personnel for administration.

These selected inclusion criteria ensured that the identified scales would be relevant and accessible to arts and health practitioners (e.g., scales that can be self-administered).

Following the procedure used in Ilya et al. (13), we then identified the top five most widely used scales that met the inclusion criteria above.

### 2.2 Frequency of scales in the top 18 arts and culture domains

In part two, we sought to capture the use of each scale across the art domains. This provides information about which scales are preferred in which arts domains, and whether the usage trends of scales are similar to the general prevalence of the scales in research within the broader health and psychological sciences.

In this search (see details in Figure 1), each of the top four frequently used scales identified in part one was assessed for frequency of use in 18 arts domains. These 18 arts domains were drawn from Fancourt and Finn (2)’s definition of the arts, and from the forms of art identified by Sonke et al. in their work on arts participation for Public Health Research (14). These 18 art forms include: performing arts, arts, music, dance, theatre, singing, visual arts, crafts, painting, literary festivals, poetry, electronic arts, gallery, museum, choir, film, concerts, and storytelling.

Following Ilya et al. we used PubMed and Google Scholar to examine the popularity of each of the top scales across the 18 arts domains (Table 1). Focusing on articles published between 1 January 2013 and 1 July 2024, we searched for the name of the scale and each arts domain. The search strategies used for retrieving the review articles on QoL in Part One and search results of QoL across 18 selected domains in Part Two are found in Figures 1A, B, respectively. An example of this search query would be: “EQ-5D” AND “Music.” A heat map was used to illustrate the frequency of the scales’ usage for each art domain on PubMed (Figure 3) and Google Scholar (Figure 4).

**Table 1:** Scales for Quality of Life.

Scales	Frequency in review paper	Purpose	Time to administer/min	No. of items	Domain/dimensions covered	Levels of measurement	Recall period	Cost
Euro-QoL(EQ-5D-3L/5L)	11	Serves to compare value health-related QoL based on weighted importance rated by user	~2–5	5	Mobility, Self-care, Usual activities, Pain/Discomfort, Anxiety/Depression	Ordinal (range 0–100)	Recent (Current state)	Copyrighted (free for non-commercial and academic use by request)
SF-36	9	Serves to quantify the state of health and measure QoL related to health. It includes fundamental human values pertinent to QoL and wellbeing by considering individual domains and incorporating 2 global components.	~10–15	36	Physical functioning, Role-physical, Bodily pain, General health, Vitality (energy/fatigue), Social functioning, Role-emotional, Mental health Each of these domains contribute to the physical and mental component summary	Ordinal (range 0–100)	Past 4 weeks (except for general health)	Copyrighted; free to use if users agree to terms and conditions
SF-12	5	Evaluates the influence of health on an individual’s daily life	~2–5	12	Same number of domains (8) as SF-36, but offers an abridged version with fewer items (questions) in each domain.	Ordinal (range 0–100)	Past 4 weeks	Copyrighted, fee waiver is possible for requested academic projects
WHOQOL-BREF	4	Assesses QoL in the context of personal culture, values, personal goals, standards, and concerns	~5–10	26	Physical health, psychological health, social relationships, environment, and two items on overall QoL and general health	Ordinal (range 0–100)	Past 2 weeks	Free

**Figure 3:**
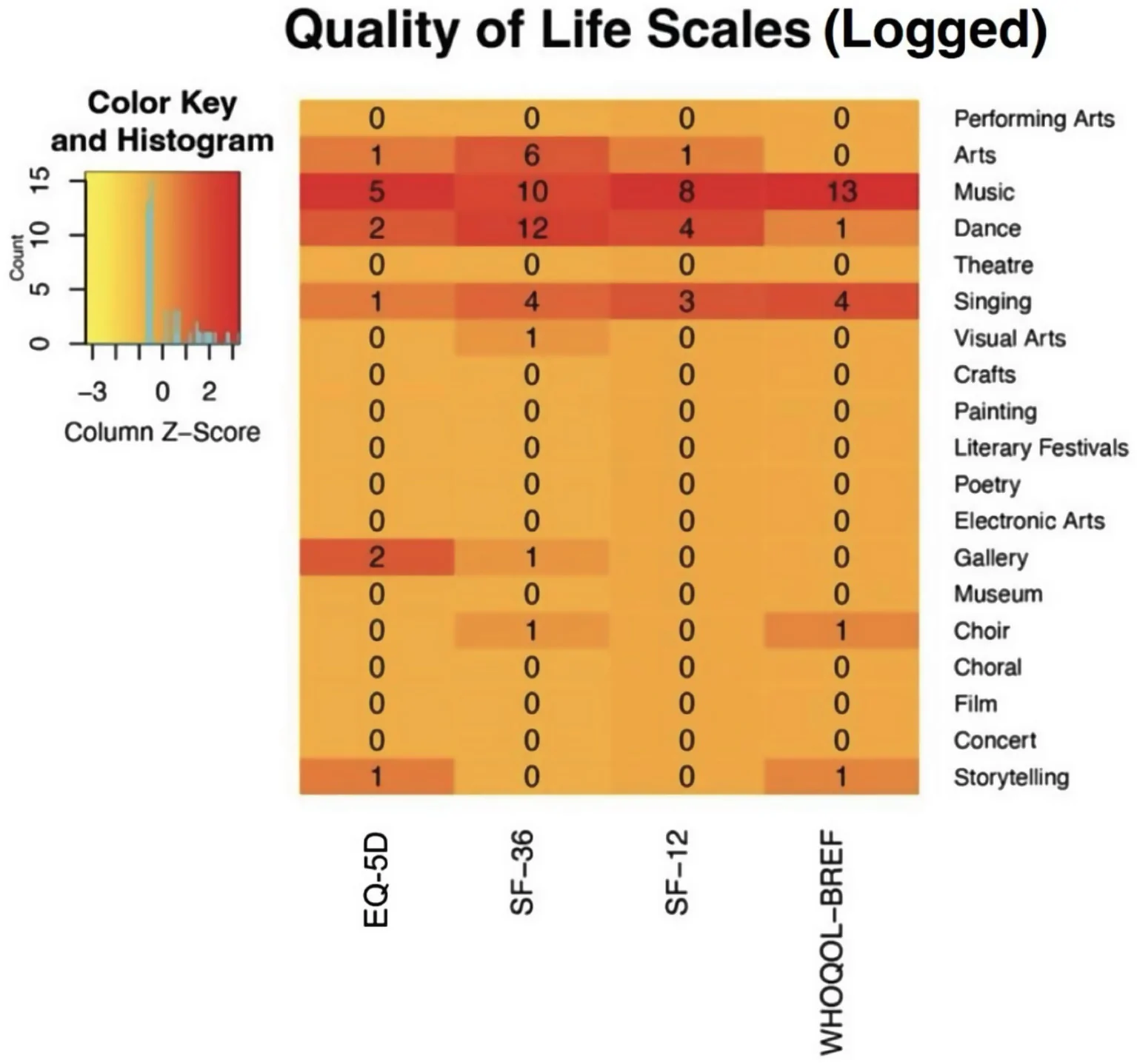
Heat Map of Quality of Life Scales from PubMed search results.

**Figure 4:**
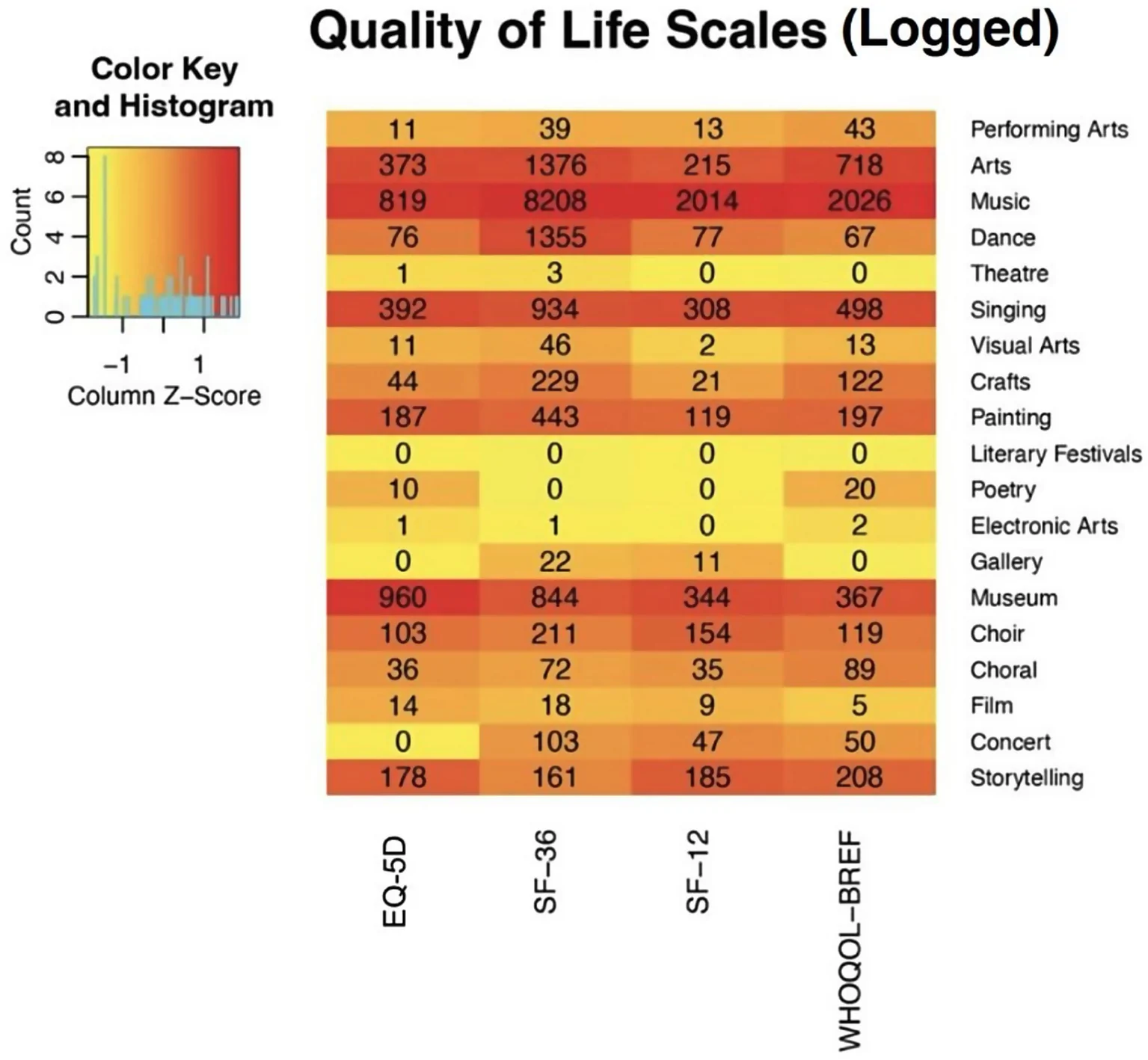
Heat Map of Quality of Life Scales from Google scholar search results.

## 3 Results

### 3.1 Number of articles and scales retrieved for QoL

In Part 1, we retrieved 149 articles mentioning QoL from the two journal collections on both PubMed and Scopus. After removing 8 duplicates and excluding 9 papers after screening the titles and abstracts, 132 articles remained. As one full-text was unretrievable, 131 full-texts were assessed for eligibility. From these, 42 articles were eligible and included in the study (see Supplementary File S2). The identification, screening, and eligibility process for Quality of Life review papers is illustrated in *PRISMA Diagram* (see Figure 2).

Upon review of these eligible papers, 71 QoL scales were identified. Of these 71 scales, 1 was excluded, as it is not available in English. We then screened the scales based on the inclusion criteria. 38 scales were found to be for specific clinical populations, 14 were for specific age groups, and 1 was excluded for being country-specific. This left a total of 17 scales that met our inclusion criteria, all mentioned more than twice in the reviews.

From these 17 scales that met our criteria, the top four most frequently-used scales are: EuroQol Five-Dimension Three-Level/Five-Level (EQ-5D-3L/5L), 36-Item Short Form Health Survey (SF-36), World Health Organization Quality of Life-BREF (WHOQOL-BREF), and 12-Item Short Form Health Survey (SF-12) (in descending order). Their characteristics are summarised in Table 1 on Scales for Quality of Life.

As multiple scales tied for fifth place (the Quality of Life Inventory [QOLI], Assessment of Quality of Life, Manchester Short Assessment of Quality of Life [MANSA], and the Quality of Life Enjoyment and Satisfaction Questionnaire [Q-LES-Q]), only the top four scales were included in Part Two of this paper, consistent with our aim to include up to five of the most frequently cited scales.

### 3.2 Characteristics of QoL scales

The four QoL scales have a range of 5 to 36 items, and the total administration time of the scales ranges between 2 and 10 min. Of the four scales, EQ-5D and SF-12 can be completed the fastest (2–5 min). WHOQOL-BREF takes 5–10 min to be completed, and SF-36 takes the longest at 10–15 min. SF-36’s longer administration time is likely attributable to its comprehensive measurement of QoL with 36 items in its scale, the longer version of SF-12. As the abridged version of the Short Form Health Survey, SF-12 has fewer questions per domain compared to SF-36. It provides good ground coverage for an elementary assessment of QoL, but lacks the specificity and depth in each domain that could be more useful in nuanced studies.

Among the four scales, EQ-5D is very brief and easy to use across diverse populations. It covers five domains: mobility, self-care, usual activities, pain/discomfort, and anxiety/ depression. It has broader coverage compared with SF-36, which includes Physical functioning, Role-physical, Bodily pain, General health, Vitality (energy/fatigue), Social functioning, Role-emotional, and Mental health. Compared to EQ-5D, SF-36 also has more items per domain, each with a wide score range of 0–100. This makes SF-36 a sensitive scale that provides comprehensive coverage across multiple domains. SF-12 is the abridged version of SF-36 with fewer questions per domain. While it does not provide as in-depth an analysis of an individual’s QoL, it offers quicker, elementary coverage in assessing QoL. In comparison, WHOQOL-BREF is designed for the assessment of self-perceived Quality of Life in the general population, and has physical, psychological, social, and environmental domains. It was designed to be suitable for evaluations in different cultural settings, and is a popular choice when conducting cross-cultural comparisons.

In terms of cost, SF-12, SF-36, and EQ-5D all require a license for usage. EQ-5D is free upon request if used in non-commercial academic contexts, and WHOQOL-BREF is completely free for use. Digital copies of all four QoL instruments are available in multiple languages including Mandarin, Malay, Tamil, and others, although access and reproduction of the SF-12, SF-36, and EQ-5D are subject to their respective licensing conditions (11).

Time-frame is another key consideration, as a scale’s recall period can affect an individual’s reporting of their QoL. This would affect the processing and interpretation of the data, and must be evaluated across different scales. Of the 4 scales that are analysed, EQ-5D does not have a specific recall period, and is based on an individual’s current state. Comparatively, SF-36 and SF-12 have a 4-week recall period, while WHOQOL-BREF has a 2-week recall period.

The characteristics of QoL scales are summarised in Table 1 on Scales for Quality of Life.

### 3.3 QoL scales’ popularity across arts domains

In the second phase of the review, we quantified the frequency of each scale’s use in research articles across 18 arts domains, using publication counts derived from PubMed and Google Scholar searches. This is summarised in the two heat maps presented in Figures 3, 4.

In both databases, similar trends can be observed for the scales’ popularity within the arts: SF-36 yielded the highest total number of articles retrieved, followed by WHOQOL-BREF, SF-12, and then EQ-5D.

Across all four scales, music yielded the highest number of articles, indicating a more extensive QoL evidence base compared to the other arts domains.

Further, in PubMed, there were several domains with no articles retrieved (Figure 3). These included Performing Arts, Theatre, Literary Festivals, Poetry, Electronic Arts, Museum, Choral, Film, and Concert. In Google Scholar, only Literary Festivals yielded zero results. As presented in Figures 3, 4, Theatre, Electronic Arts and Literary Festivals all have the lowest number of articles retrieved across at least 3 of the top 4 scales.

## 4 Discussion

This paper aims to provide recommendations for pre-existing, frequently used scales measuring Quality of Life for arts and health practitioners. These recommendations are designed for practitioners and informed by a two-part literature review to guide the evaluation of community arts interventions that aim to improve health outcomes.

### 4.1 Prevalence of QoL scale usage

In the first part of the study, we identified the most frequently used Quality of Life tools within the health and psychological science literature, namely: Euro-QoL (EQ-5D-3L/5L) EQ-5D, SF-36, WHOQOL-BREF, and SF-12 (in descending order).

Although the EQ-5D was the top choice in health and psychological sciences, the second part of the study revealed that this was ranked fourth within the published literature for the arts. By contrast, QoL research for the arts favoured SF-36. In Section 4.2, we elaborate on the properties of each QoL scale and compare how art-specific considerations may influence the choice of tools.

Across the 18 art domains studied, we further observed that for most QoL scales, music yielded the highest number of articles. This suggests that music is most widely studied in relation to Quality of Life—a pattern broadly consistent with the wider literature on arts and health. These trends may reflect the near-universal accessibility of music and its well-documented impact on emotion (15–18).

For the EQ-5D measure, the museum domain yielded the largest number of articles on Google Scholar. It also yielded the second most articles (after music) for SF-12. Studies involving these scales included: evaluations of how a museum visit may impact Quality of Life (see, for example, the benefits of physician-prescribed museum visit by Beauchet et al. and Kluge-Pinsker and Stauffer (19, 20), or the impact of art-based activities within museums (21). The relative popularity of measuring Quality of Life in these settings may point to a growing mandate for institutions to direct their services toward improving audience wellbeing. Still, these initiatives are relatively nascent, and more systematic, quantitative, and larger-scale assessments are needed.

Finally, across all scales and search databases, the art domains with the fewest QoL studies were literary festivals and electronic arts. Rosetti argues that although literary festivals show promise in terms of positively impacting their audience, the wellbeing outcomes of literary festivals on attendees are relatively underinvestigated (22). In the case of electronic arts, this may be due to its status as a relatively newer art form. Still, with the increased accessibility that technology brings and its potential for wide-reaching impact, we believe it will be a promising field for arts interventions. The online sphere may engage audiences differently, and when electronic art interventions gain popularity, future work could evaluate the most suitable scales for evaluating QoL in these contexts.

### 4.2 Evaluation of scale characteristics and recommendations

Section 4.1 highlighted the most widely-used QoL scales in health and psychological science, and discussed how patterns of scale usage: (i) differed from those in art-based research, and (ii) across art domains. Below, we discuss the characteristics of each QoL scale, compare/contrast the scales’ recall period, and explore how scale characteristics may influence selection in art evaluations.

#### EQ-5D

First, we note that EQ-5D is widely popular - both in the broader health and psychological literature, and in research pertaining to museums. The overall high use of EQ-5D may be attributed to its easy administration: it has a relatively short administration time, the least number of items, and gauges overall QoL of an individual by assessing five dimensions of health. It is often used for a quick snapshot or as a brief diagnostic tool.

Studies with multiple collection points would benefit from a scale like EQ-5D because its easy administration increases participant compliance, reduces questionnaire fatigue, and helps to minimise attrition, thereby supporting data adequacy and accuracy. These practical advantages make EQ-5D well-suited to repeated-measures designs, and may therefore account for its use in Beauchet et al. (23), which measured the effects of art museum visits on the QoL of dwelling adults at six different timepoints following the pre-intervention baseline.

Although EQ-5D’s brevity makes it well-suited for routine clinical and diagnostic use, it may be less sensitive to the nuanced and potentially multidimensional effects of arts interventions. Therefore, we recommend that practitioners review and compare the different domains assessed by each scale as part of their scale selection decision-making process. The domains covered by each scale are summarised in Table 1 and discussed below.

#### SF-36 and SF-12

Unlike EQ-5D, which covers 5 domains of physical and mental health, SF-36 goes deeper by including social functioning and vitality. It accounts for variations in both physical and mental health and, with its wider score range, offers greater multidimensional sensitivity, especially as compared to EQ-5D. This could explain why SF-36 was found to be more popular in its usage across various arts domains – it is a lengthier version of SF-12 (36 rather than 12 items) that provides more detailed data across domains of QoL that are potentially relevant to arts interventions. Notably, Bougiesi and Zisi (24) used the Greek SF-36 to assess the QoL in young adults engaged in dance and exercise, mapping domain scores against physical activity levels to examine how activity differentially influences specific QoL dimensions rather than just overall QoL.

#### WHOQOL-BREF

WHOQOL-BREF was specifically designed by the World Health Organization to allow cross-cultural, multidimensional, and internationally-comparable evaluation of Quality of Life. It is also suitable for assessing QoL in terms of psychological health, social relationships, physical health, and environment. Similar to SF-36, WHOQOL-BREF is not a quick scale used for diagnostic purposes; rather, it may be employed to understand the relative contribution of the aforementioned areas to overall QoL. For example, a study by Jalambadni et al. used WHOQOL-BREF to assess the effectiveness of mindfulness-based art therapy on women with breast cancer, and demonstrated significant improvements in the key aspects of Quality of Life, including all four domains mentioned above (25).

#### Recall period

Recall period is another important consideration, and may depend on the nature and expected impact of the arts intervention. SF-36 is a scale with a longer recall period of 4 weeks, while EQ-5D considers an individual’s current state at the point of administration. By capturing average health and functioning over a longer timeframe, measures are less influenced by short-term fluctuations, and are therefore well-suited for longitudinal evaluations of multi-session arts programmes, such as the study by Ellis-Hill et al. (26).

Similarly, WHOQOL-BREF has a relatively long recall period of 2 weeks, which is used to capture recent lived experience, rather than momentary Quality of Life. Compared to SF-12 and SF-36, WHOQOL-BREF may be less susceptible to long-term recall bias, as it has a 2-week rather than a 4-week recall period.

In contrast, scales such as EQ-5D measure the individual’s current state at the point of administration, making it more suitable in capturing the immediate, post-intervention effects that may be more applicable to single-session arts programmes. Sanghera et al. (27) supports this argument through their study examining the influence of recall period on reporting outcomes when using SF-12 as compared to EQ-5D. The timing and recall period of these 2 scales were found to have significantly affected the reported quality-adjusted life-years in chemotherapy patients with fluctuating health. EQ-5D was able to pick up the differences much more clearly, and Sanghera et al. thus suggests capturing QoL by using a scale with a brief recall period when fluctuations are likely.

### 4.3 Summary of relevant considerations

We recommend that arts practitioners match their program duration with the scales’ recall time frame for accurate and meaningful results, and consider the number of times a scale will be administered through multi-session programmes. This is summarised in Table 1.

Although administration time is an important consideration given the limited time investigators have for evaluation (28), our findings suggest that other factors may play a larger role in scale selection. For example, despite having the longest administration time, SF-36 was found to be the most popular scale amongst the arts domains in Part 2 of our study. This may reflect its sensitivity across multiple QoL domains and its no-cost use upon agreement to its terms and conditions, making it an attractive choice for arts practitioners seeking comprehensive QoL evaluation within budget constraints. As a further illustration of the role of cost, SF-12 was the least popular among the top 4 QoL scales, potentially because it is under copyright and comes with a fee. While cost is not, of course, the sole factor influencing scale popularity, these findings suggest that researchers, including arts practitioners, may prioritise the depth and quality of evaluation over speed and efficiency, in addition to budget considerations. Further research is still needed to confirm this claim because all the scales in this review are relatively brief and take at most 15 min to complete.

Careful consideration of the domains a scale covers, as well as the specific items it includes, is essential in any intervention study. This is particularly important in arts interventions targeting QoL, as QoL is a broad and multidimensional construct with different operationalisations across instruments and subfields. Work by Costa et al. and others acknowledges the ambiguity and heterogeneity that remain in defining QoL universally, and has attempted to delineate its parameters (29). Definitions and interpretations of Quality of Life are shaped by cultural and value systems (30), and may therefore differ across societies in terms of what is considered a meaningful, fulfilling, life well-lived. Given that arts interventions may affect various aspects of QoL, it is important to consider the scope (e.g., domains covered) of the measurement tool. Arts practitioners should therefore select the scale that is most appropriate and sensitive to the specific changes their intervention intends to produce.

### 4.4 Limitations

Since QoL is a multi-dimensional health outcome woven into many areas of life, future work could involve an exploration of articles beyond the areas of mental health and psychological science that this review focused on for the retrieval of reviews and scales.

The Arts are diverse, and this review makes recommendations for more commonly used programme structures, with considerations and constraints that are commonly encountered by arts practitioners. Future studies could examine additional outcome measures, a broader range of art forms, and specific demographic or clinical populations of growing interest. Future work could also evaluate the use of existing scales in the context of heritage activities, with the aim of tailoring assessment approaches to the needs of subcultures in ways that advance health while preserving local arts and historical context.

## 5 Conclusion

Our findings indicate that EQ-5D is the most widely used QoL scale overall, whereas the SF-36 is most frequently used within arts and cultural domains. The prominence of the SF-36 in arts contexts may reflect its multidimensional sensitivity, which may be well-suited to capture the complex and varied outcomes targeted by many arts interventions. In contrast, EQ-5D may be better suited as a quick diagnostic clinical scale, given its shorter administration time and assessment data.

Although this review evaluates scale popularity across arts domains, selection should ultimately be guided by contextual fit. Practitioners should consider the strengths and limitations of each scale in relation to their intervention goals, population, duration, and budget. For interventions with financial constraints, the SF-36 and EQ-5D may be especially practical options. The longer recall periods of the SF-36 and SF-12 make them more appropriate for multi-session programmes requiring longer-term evaluation, while the WHOQOL-BREF offers strong cross-cultural applicability, facilitating comparisons across cultural settings. Among the reviewed scales, the SF-36 provides the most comprehensive coverage of QoL domains, and may therefore be particularly suitable for arts practitioners seeking a more in-depth and nuanced evaluation of their intervention’s impact.
