# Supplementary material for: Towards recommendations for arts and health evaluation: a two-part review of Quality of Life scales used in mental health and arts research
Source: Front Public Health. 2026 Oct 12;14:1933623. doi: 10.3389/fpubh.2026.1933623 (PMC13700918; doi:10.3389/fpubh.2026.1933623)
Supplement: Supplementary file 2 [file Supplementary_file_2.DOCX]

**Supplementary File S2: Table of Review Papers included for Quality-of-Life Scales**

| Scales Mentioned for Quality of Life | Title | Authors | Journal/Book | Year Published |
| --- | --- | --- | --- | --- |
| EQ-5D, WHOQOL-BREF, SF-12, | Psychological therapies for women who experience intimate partner violence | Hameed M, O'Doherty L, Gilchrist G, Tirado-Muñoz J, Taft A, Chondros P, Feder G, Tan M, Hegarty K. | Cochrane Database Syst Rev | 2020 |
| AAQoL, AIM-A, AIM-C, CHIP-CE, CHQ, KINDL-R,  Q-LES-Q-SF,  YQOL-R, PedsQL | Systematic Review and Meta-Analysis: Effects of Pharmacological Treatment for Attention-Deficit/Hyperactivity Disorder on Quality of Life | Bellato A, Perrott NJ, Marzulli L, Parlatini V, Coghill D, Cortese S. | J Am Acad Child Adolesc Psychiatry | 2024 |
| WHOQOL-BREF, European Organization for Research and Treatment of Cancer Quality of Life Questionnaire-C30 (EORTC QLQ-C30) | A systematic review of the measurement properties of the Body Image Scale (BIS) in cancer patients | Melissant HC, Neijenhuijs KI, Jansen F, Aaronson NK, Groenvold M, Holzner B, Terwee CB, van Uden-Kraan CF, Cuijpers P, Verdonck-de Leeuw IM. | Support Care Cancer | 2018 |
| EORTC QLQ-C30, EORTC QLQ-CR38, EORTC QLQ-CR29 | Measuring health-related quality of life in colorectal cancer patients: systematic review of measurement properties of the EORTC QLQ-CR29 | van der Hout A, Neijenhuijs KI, Jansen F, van Uden-Kraan CF, Aaronson NK, Groenvold M, Holzner B, Terwee CB, van de Poll-Franse LV, Cuijpers P, Verdonck-de Leeuw IM. | Support Care Cancer | 2019 |
| PedsQL | Quality of life in children: A critical examination of concepts, approaches, issues, and future directions | Wallander J.L., Koot H.M. | Clinical Psychology Review | 2016 |
| PQ-LES-Q, MANSA(Manchester Short Assessment of Quality of Life) | Systematic Review and Meta-Analysis: Cognitive-Behavioral Therapy for Obsessive-Compulsive Disorder in Children and Adolescents | Uhre C.F., Uhre V.F., Lønfeldt N.N., Pretzmann L., Vangkilde S., Plessen K.J., Gluud C., Jakobsen J.C., Pagsberg A.K. | Journal of the American Academy of Child and Adolescent Psychiatry | 2020 |
| 15D, 16D, 17D, EQ-5D-3L, EQ-5D-5L, EQ-5D-Y | Patient-reported outcome measures suitable for quality of life/well-being assessment in multisectoral, multinational and multiperson mental health economic evaluations | Łaszewska A., Helter T.M., Nagel A., Perić N., Simon J. | Evidence-Based Mental Health | 2022 |
| Euro-QoL, SF-12, QOLI | Systematic review and meta-analysis of transdiagnostic psychological treatments for anxiety and depressive disorders in adulthood | Newby J.M., McKinnon A., Kuyken W., Gilbody S., Dalgleish T. | Clinical Psychology Review | 2015 |
| Q-LES-Q, SF-36, EQ-5D, QOLI | The clinical characterization of the adult patient with an anxiety or related disorder aimed at personalization of management | Stein D.J., Craske M.G., Rothbaum B.O., Chamberlain S.R., Fineberg N.A., Choi K.W., de Jonge P., Baldwin D.S., Maj M. | World Psychiatry | 2021 |
| WHODAS 2.0-12, IWQOL-Lite, EDQOL, EDQLS, HERQpLED, QOL-ED, SF-12, SF-6D,EQ-5D-5L, HRQOL-4, PedsQL 4.0 SF-15, QoL Scale | International consensus on patient-centred outcomes in eating disorders | Austin A., De Silva U., Ilesanmi C., Likitabhorn T., Miller I., Sousa Fialho M.D.L., Austin S.B., Caldwell B., Chew C.S.E., Chua S.N., Dooley-Hash S., Downs J., El Khazen Hadati C., Herpertz-Dahlmann B., Lampert J., Latzer Y., Machado P.P.P., Maguire S., Malik M., Moser C.M., Myers E., Pastor I.R., Russell J., Smolar L., Steiger H., Tan E., Trujillo-Chi Vacuán E., Tseng M.-C.M., van Furth E.F., Wildes J.E., Peat C., Richmond T.K. | The Lancet Psychiatry | 2023 |
| SF-6D, EQ-5D-5L | Perinatal mental health: a review of progress and challenges | Howard L.M., Khalifeh H. | World Psychiatry | 2020 |
| WHOQOL-BREF, Q-LES-Q, SQLS | Self-help interventions for psychosis: A meta-analysis | Scott A.J., Webb T.L., Rowse G. | Clinical Psychology Review | 2015 |
| MANSA, QOLI | Instruments for measuring mental health recovery: A systematic review | Sklar M., Groessl E.J., O'Connell M., Davidson L., Aarons G.A. | Clinical Psychology Review | 2013 |
| EUROQOL | The efficacy of short-term psychodynamic psychotherapy for depression: A meta-analysis update | Driessen E., Hegelmaier L.M., Abbass A.A., Barber J.P., Dekker J.J.M., Van H.L., Jansma E.P., Cuijpers P. | Clinical Psychology Review | 2015 |
| PedsQL | Conceptual review of measuring functional impairment: Findings from the Weiss Functional Impairment Rating Scale | Weiss M.D., McBride N.M., Craig S., Jensen P. | Evidence-Based Mental Health | 2018 |
| SF-36 | Psychiatric and neuropsychiatric presentations associated with severe coronavirus infections: a systematic review and meta-analysis with comparison to the COVID-19 pandemic | Rogers J.P., Chesney E., Oliver D., Pollak T.A., McGuire P., Fusar-Poli P., Zandi M.S., Lewis G., David A.S. | The Lancet Psychiatry | 2020 |
| St. George’s Respiratory Questionnaire, Chronic Respiratory Questionnaire, SF-36 | Efficacy of psychosocial interventions on psychological and physical health outcomes in chronic obstructive pulmonary disease: A systematic review and meta-analysis | Farver-Vestergaard I., Jacobsen D., Zachariae R. | Psychotherapy and Psychosomatics | 2015 |
| WHOQOL | The clinical characterization of the patient with primary psychosis aimed at personalization of management | Maj M., van Os J., De Hert M., Gaebel W., Galderisi S., Green M.F., Guloksuz S., Harvey P.D., Jones P.B., Malaspina D., McGorry P., Miettunen J., Murray R.M., Nuechterlein K.H., Peralta V., Thornicroft G., van Winkel R., Ventura J. | World Psychiatry | 2021 |
| GTS-QOL | A personal 35 year perspective on Gilles de la Tourette syndrome: Assessment, investigations, and management | Robertson M.M. | The Lancet Psychiatry | 2015 |
| QOL-AD, QUALID, WHO-QOL-OLD | Artificial intelligence for older people receiving long-term care: a systematic review of acceptability and effectiveness studies | Loveys K., Prina M., Axford C., Domènec Ò.R., Weng W., Broadbent E., Pujari S., Jang H., Han Z.A., Thiyagarajan J.A. | The Lancet Healthy Longevity | 2022 |
| Stroke Specific QoL Scale, Stroke Impact Scale | Yoga for stroke rehabilitation | Lawrence M, Celestino Junior FT, Matozinho HH, Govan L, Booth J, Beecher J. | Cochrane Database Syst Rev | 2017 |
| EQ-5D | The impact of Otago exercise programme on the prevention of falls in older adult: A systematic review | Yang Y, Wang K, Liu H, Qu J, Wang Y, Chen P, Zhang T, Luo J. | Front Public Health | 2022 |
| Asthma Quality of Life Questionnaire | Effects of exercise-based pulmonary rehabilitation on adults with asthma: a systematic review and meta-analysis | Feng Z, Wang J, Xie Y, Li J. | Respir Res | 2021 |
| PWD-QOL(**Quality of Life for Persons with Dementia)**, Quality of Life in Alzheimer's Disease (QoL-AD) | Quality of family relationships and outcomes of dementia: a systematic review | Edwards HB, Ijaz S, Whiting PF, Leach V, Richards A, Cullum SJ, Cheston RI, Savović J. | BMJ Open | 2018 |
| Dutch RAND-36 HRQoL, SF-8, EORTC QLQ-C30, The Functional Assessment of Cancer Therapy – General (FACT-G) | Quality of Life in Adult Cancer Patients With Underage Children: An Integrative Review | Mota-George G, Martinez A, Dains JE. | J Adv Pract Oncol | 2023 |
| INQoL, SF-36, LIFE-H, SIP | A Review of Psychopathology Features, Personality, and Coping in Myotonic Dystrophy Type 1 | Minier L, Lignier B, Bouvet C, Gallais B, Camart N. | J Neuromuscul Dis | 2018 |
| SF-36 | The impact of Exergames on emotional experience: a systematic review | Marques LM, Uchida PM, Barbosa SP. | Front Public Health | 2023 |
| SF-12 | Positive psychological well-being and clinical characteristics in IBS: A systematic review | Madva EN, Sadlonova M, Harnedy LE, Longley RM, Amonoo HL, Feig EH, Millstein RA, Zambrano J, Rojas Amaris A, Jurayj J, Burton Murray H, Staller K, Kuo B, Keefer L, Huffman JC, Celano CM. | Gen Hosp Psychiatry | 2023 |
| SF-36 | Acupuncture Treatment for Emotional Problems in Women with Infertility: A Systematic Review and Meta-Analysis | Hwang SI, Yoon YJ, Sung SH, Cho SJ, Park JK. | Healthcare (Basel) | 2023 |
| PedsQL | Emotion Regulation Flexibility and Electronic Patient-Reported Outcomes: A Framework for Understanding Symptoms and Affect Dynamics in Pediatric Psycho-Oncology | Mirzaie K, Burns-Gebhart A, Meyerheim M, Sander A, Graf N. | Cancers (Basel) | 2022 |
| EQ-5D, SF-36, CRQ | Significance of Pulmonary Rehabilitation in Improving Quality of Life for Subjects With COPD | Yang J, Lin R, Xu Z, Zhang H. | Respir Care | 2019 |
| IBS-QOL, SF-36 | Self-Help for Medically Unexplained Symptoms: A Systematic Review and Meta-Analysis | van Gils A, Schoevers RA, Bonvanie IJ, Gelauff JM, Roest AM, Rosmalen JG. | Psychosom Med | 2016 |
| IBS-QOL, IBD-QOL, DAS, SF-36, SCL-90-R | Coping Strategies and Irritable Bowel Syndrome: A Systematic Review | David L, Fadgyas Stanculete M, Bolba AR, Chiaroni G, Barsan M, Popa SL. | J Gastrointestin Liver Dis | 2021 |
| SGRQ, CCQ, CRQ | Effectiveness of motivational interviewing among patients with COPD: A systematic review with meta-analysis and trial sequential analysis of randomized controlled trials | Wang C, Liu K, Sun X, Yin Y, Tang T. | Patient Educ Couns | 2022 |
| SGRQ, HRQoL, CRQ | Efficacy of unsupervised exercise in adults with obstructive lung disease: a systematic review and meta-analysis | Taylor D, Jenkins AR, Parrott K, Benham A, Targett S, Jones AW. | Thorax | 2021 |
| WHOQOL-BREF, EQ-5D, | Psychological interventions for diabetes-related distress in adults with type 2 diabetes mellitus | Chew BH, Vos RC, Metzendorf MI, Scholten RJ, Rutten GE. | Cochrane Database Syst Rev | 2017 |
| Dermatology Life Quality Index (DLQI), Women’s Androgenetic Alopecia Quality of Life index, WHOQOL-BREF, Dermatology Quality of Life instrument in Turkish , Modified Women’s Androgenetic Alopecia Quality of Life index | Health-Related Quality of Life, Depression, and Self-esteem in Patients With Androgenetic Alopecia: A Systematic Review and Meta-analysis | Huang CH, Fu Y, Chi CC. | JAMA Dermatol | 2021 |
| Seventeen-dimensional measure of HRQoL(17D)  Assessment of Quality of Life 6-Dimension (AQoL-6D)  Health Utilities Index 2(HUI2)  Health Utilities Index 3(HUI3)  EQ VAS  Quality of Wellbeing(QWB)  Eq-5D  EQ-5D-3L  EQ-5D-Y-3L  Adolescent Health Utility Measure (AHUM)  Eq-5d—Y5L  Child Health Utility 9 Dimensions(CHU9D) | Are We Agreed? Self- Versus Proxy-Reporting of Paediatric Health-Related Quality of Life (HRQoL) Using Generic Preference-Based Measures: A Systematic Review and Meta-Analysis | Khanna D, Khadka J, Mpundu-Kaambwa C, Lay K, Russo R, Ratcliffe J; Quality of Life in Kids: Key Evidence to Strengthen Decisions in Australia (QUOKKA) Project Team. | Pharmacoeconomics | 2022 |
| SF-36 short form, Thyroid surgery-specific questionnaire, thyroid cancer-specific quality of life questionnaire (THYCA-QOL), SF-12 short-form, General Quality of Life Inventory (GQOLI-74) | Advances in the assessment of cosmetic outcomes, sensory alteration in surgical areas, and health-related quality of life of endoscopic thyroidectomy | Zhou D, Zhang Z, Dou X, Xia F, Li X. | World J Surg Oncol | 2024 |
| 8-disease Parkinson's Disease Questionnaire (PDQ-8) | Rotigotine transdermal patch for the treatment of neuropsychiatric symptoms in Parkinson's disease: A meta-analysis of randomized placebo-controlled trials | Wang HT, Wang L, He Y, Yu G. | J Neurol Sci | 2018 |
| Child Health Utility 9D (CHU9D), QWB, EQ-5D-Y, AHUM, AQOL-6D, HRQOL_17D | Mind the (inter-rater) gap. An investigation of self-reported versus proxy-reported assessments in the derivation of childhood utility values for economic evaluation: A systematic review | Khadka J, Kwon J, Petrou S, Lancsar E, Ratcliffe J. | Soc Sci Med | 2019 |
| Migraine-Specific Quality of Life Questionnaire | Effect of acupuncture on emotional disorder in patients with chronic pain: a Meta-analysis | Sun N, Zhang N, Lin LL, Wang LQ, Fan H, Yang JW, Liu CZ. | Zhongguo Zhen Jiu | 2020 |

**Reference List of Included Review Papers from order of appearance in the above table**

1. Hameed M, O’Doherty L, Gilchrist G, Tirado-Muñoz J, Taft A, Chondros P, et al. Psychological therapies for women who experience intimate partner violence. Cochrane Database Syst Rev [Internet]. 2020 Jul 1;2020(7). Available from: https://doi.org/10.1002/14651858.cd013017.pub.
2. Bellato A, Perrott NJ, Marzulli L, Parlatini V, Coghill D, Cortese S. Systematic review and meta-analysis: effects of pharmacological treatment for attention-deficit/hyperactivity disorder on quality of life. J Am Acad Child Adolesc Psychiatry [Internet]. 2024 May 1; Available from: https://doi.org/10.1016/j.jaac.2024.05.023.
3. Melissant HC, Neijenhuijs KI, Jansen F, Aaronson NK, Groenvold M, Holzner B, et al. A systematic review of the measurement properties of the Body Image Scale (BIS) in cancer patients. Support Care Cancer [Internet]. 2018 Mar 12;26(6):1715-26. Available from: https://doi.org/10.1007/s00520-018-4145-x.
4. Van Der Hout A, Neijenhuijs KI, Jansen F, Van Uden-Kraan CF, Aaronson NK, Groenvold M, et al. Measuring health-related quality of life in colorectal cancer patients: systematic review of measurement properties of the EORTC QLQ-CR29. Support Care Cancer [Internet]. 2019 Apr 13;27(7):2395-412. Available from: https://doi.org/10.1007/s00520-019-04764-7.
5. Wallander JL, Koot HM. Quality of life in children: a critical examination of concepts, approaches, issues, and future directions. Clin Psychol Rev [Internet]. 2015 Nov 27;45:131-43. Available from: https://doi.org/10.1016/j.cpr.2015.11.007.
6. Uhre CF, Uhre VF, Lønfeldt NN, Pretzmann L, Vangkilde S, Plessen KJ, et al. Systematic review and meta-analysis: cognitive-behavioral therapy for obsessive-compulsive disorder in children and adolescents. J Am Acad Child Adolesc Psychiatry [Internet]. 2019 Oct 4;59(1):64-77. Available from: https://doi.org/10.1016/j.jaac.2019.08.480.
7. Łaszewska A, Helter TM, Nagel A, Perić N, Simon J. Patient-reported outcome measures suitable for quality of life/well-being assessment in multisectoral, multinational and multiperson mental health economic evaluations. Evid Based Ment Health [Internet]. 2021 Dec 23;25(2):85-92. Available from: https://doi.org/10.1136/ebmental-2021-300334.
8. Newby JM, McKinnon A, Kuyken W, Gilbody S, Dalgleish T. Systematic review and meta-analysis of transdiagnostic psychological treatments for anxiety and depressive disorders in adulthood. Clin Psychol Rev [Internet]. 2015 Jun 7;40:91-110. Available from: https://doi.org/10.1016/j.cpr.2015.06.002.
9. Stein DJ, Craske MG, Rothbaum BO, Chamberlain SR, Fineberg NA, Choi KW, et al. The clinical characterization of the adult patient with an anxiety or related disorder aimed at personalization of management. World Psychiatry [Internet]. 2021 Sep 9;20(3):336-56. Available from: https://doi.org/10.1002/wps.20919.
10. Austin A, De Silva U, Ilesanmi C, Likitabhorn T, Miller I, Da Luz Sousa Fialho M, et al. International consensus on patient-centred outcomes in eating disorders. Lancet Psychiatry [Internet]. 2023 Sep 25;10(12):966-73. Available from: https://doi.org/10.1016/S2215-0366(23)00265-1.
11. Howard LM, Khalifeh H. Perinatal mental health: a review of progress and challenges. World Psychiatry [Internet]. 2020 Sep 15;19(3):313-27. Available from: https://doi.org/10.1002/wps.20769.
12. Scott AJ, Webb TL, Rowse G. Self-help interventions for psychosis: a meta-analysis. Clin Psychol Rev [Internet]. 2015 May 21;39:96-112. Available from: https://doi.org/10.1016/j.cpr.2015.05.002.
13. Sklar M, Groessl EJ, O’Connell M, Davidson L, Aarons GA. Instruments for measuring mental health recovery: a systematic review. Clin Psychol Rev [Internet]. 2013 Aug 14;33(8):1082-95. Available from: https://doi.org/10.1016/j.cpr.2013.08.002.
14. Driessen E, Hegelmaier LM, Abbass AA, Barber JP, Dekker JJM, Van HL, et al. The efficacy of short-term psychodynamic psychotherapy for depression: a meta-analysis update. Clin Psychol Rev [Internet]. 2015 Aug 1;42:1-15. Available from: https://doi.org/10.1016/j.cpr.2015.07.004.
15. Weiss MD, McBride NM, Craig S, Jensen P. Conceptual review of measuring functional impairment: findings from the Weiss Functional Impairment Rating Scale. Evid Based Ment Health [Internet]. 2018 Oct 12;21(4):155-64. Available from: https://doi.org/10.1136/ebmental-2018-300025.
16. Rogers JP, Chesney E, Oliver D, Pollak TA, McGuire P, Fusar-Poli P, et al. Psychiatric and neuropsychiatric presentations associated with severe coronavirus infections: a systematic review and meta-analysis with comparison to the COVID-19 pandemic. Lancet Psychiatry [Internet]. 2020 May 18;7(7):611-27. Available from: https://doi.org/10.1016/S2215-0366(20)30203-0.
17. Farver-Vestergaard I, Jacobsen D, Zachariae R. Efficacy of psychosocial interventions on psychological and physical health outcomes in chronic obstructive pulmonary disease: a systematic review and meta-analysis. Psychother Psychosom [Internet]. 2014 Dec 24;84(1):37-50. Available from: https://doi.org/10.1159/000367635.
18. Maj M, Van Os J, De Hert M, Gaebel W, Galderisi S, Green MF, et al. The clinical characterization of the patient with primary psychosis aimed at personalization of management. World Psychiatry [Internet]. 2021 Jan 12;20(1):4-33. Available from: https://doi.org/10.1002/wps.20809.
19. Robertson MM. A personal 35 year perspective on Gilles de la Tourette syndrome: assessment, investigations, and management. Lancet Psychiatry [Internet]. 2015 Jan 1;2(1):88-104. Available from: https://doi.org/10.1016/S2215-0366(14)00133-3.
20. Loveys K, Prina M, Axford C, Domènec ÒR, Weng W, Broadbent E, et al. Artificial intelligence for older people receiving long-term care: a systematic review of acceptability and effectiveness studies. Lancet Healthy Longev [Internet]. 2022 Apr 1;3(4):e286-97. Available from: https://doi.org/10.1016/S2666-7568(22)00034-4.
21. Lawrence M, Celestino FT Junior, Matozinho HH, Govan L, Booth J, Beecher J. Yoga for stroke rehabilitation. Cochrane Database Syst Rev [Internet]. 2017 Dec 8;2017(12). Available from: https://doi.org/10.1002/14651858.cd011483.pub2.
22. Yang Y, Wang K, Liu H, Qu J, Wang Y, Chen P, et al. The impact of Otago exercise programme on the prevention of falls in older adult: a systematic review. Front Public Health [Internet]. 2022 Oct 20;10. Available from: https://doi.org/10.3389/fpubh.2022.953593.
23. Feng Z, Wang J, Xie Y, Li J. Effects of exercise-based pulmonary rehabilitation on adults with asthma: a systematic review and meta-analysis. Respir Res [Internet]. 2021 Jan 30;22(1). Available from: https://doi.org/10.1186/s12931-021-01627-w.
24. Edwards HB, Ijaz S, Whiting PF, Leach V, Richards A, Cullum SJ, et al. Quality of family relationships and outcomes of dementia: a systematic review. BMJ Open [Internet]. 2018 Jan 1;8(1):e015538. Available from: https://doi.org/10.1136/bmjopen-2016-015538.
25. Mota-George G, Martinez A, Dains JE. Quality of life in adult cancer patients with underage children: an integrative review. J Adv Pract Oncol [Internet]. 2023 Jul 1;14(5):390-402. Available from: https://doi.org/10.6004/jadpro.2023.14.5.4.
26. Minier L, Lignier B, Bouvet C, Gallais B, Camart N. A review of psychopathology features, personality, and coping in myotonic dystrophy type 1. J Neuromuscul Dis [Internet]. 2018 Jul 20;5(3):279-94. Available from: https://doi.org/10.3233/JND-180310.
27. Marques LM, Uchida PM, Barbosa SP. The impact of exergames on emotional experience: a systematic review. Front Public Health [Internet]. 2023 Sep 7;11. Available from: https://doi.org/10.3389/fpubh.2023.1209520.
28. Madva EN, Sadlonova M, Harnedy LE, Longley RM, Amonoo HL, Feig EH, et al. Positive psychological well-being and clinical characteristics in IBS: a systematic review. Gen Hosp Psychiatry [Internet]. 2023 Jan 11;81:1-14. Available from: https://doi.org/10.1016/j.genhosppsych.2023.01.004.
29. Hwang SI, Yoon YJ, Sung SH, Cho SJ, Park JK. Acupuncture treatment for emotional problems in women with infertility: a systematic review and meta-analysis. Healthcare [Internet]. 2023 Oct 10;11(20):2704. Available from: https://doi.org/10.3390/healthcare11202704.
30. Mirzaie K, Burns-Gebhart A, Meyerheim M, Sander A, Graf N. Emotion regulation flexibility and electronic patient-reported outcomes: a framework for understanding symptoms and affect dynamics in pediatric psycho-oncology. Cancers [Internet]. 2022 Aug 11;14(16):3874. Available from: https://doi.org/10.3390/cancers14163874.
31. Yang J, Lin R, Xu Z, Zhang H. Significance of pulmonary rehabilitation in improving quality of life for subjects with COPD. Respir Care [Internet]. 2018 Dec 21;64(1):99-107. Available from: https://doi.org/10.4187/respcare.06353.
32. Van Gils A, Schoevers RA, Bonvanie IJ, Gelauff JM, Roest AM, Rosmalen JGM. Self-help for medically unexplained symptoms: a systematic review and meta-analysis. Psychosom Med [Internet]. 2016 May 17;78(6):728-39. Available from: https://doi.org/10.1097/psy.0000000000000325.
33. David L, Stanculete MF, Bolba AR, Chiaroni G, Barsan M, Popa SL. Coping strategies and irritable bowel syndrome: a systematic review. J Gastrointestin Liver Dis [Internet]. 2021 Nov 8;30(4):485-94. Available from: https://doi.org/10.15403/jgld-3937.
34. Wang C, Liu K, Sun X, Yin Y, Tang T. Effectiveness of motivational interviewing among patients with COPD: a systematic review with meta-analysis and trial sequential analysis of randomized controlled trials. Patient Educ Couns [Internet]. 2022 Jul 28;105(11):3174-85. Available from: https://doi.org/10.1016/j.pec.2022.07.019.
35. Taylor D, Jenkins AR, Parrott K, Benham A, Targett S, Jones AW. Efficacy of unsupervised exercise in adults with obstructive lung disease: a systematic review and meta-analysis. Thorax [Internet]. 2021 Mar 8;76(6):591-600. Available from: https://doi.org/10.1136/thoraxjnl-2020-216007.
36. Chew BH, Vos RC, Metzendorf MI, Scholten RJ, Rutten GE. Psychological interventions for diabetes-related distress in adults with type 2 diabetes mellitus. Cochrane Database Syst Rev [Internet]. 2017 Sep 27;2017(10). Available from: https://doi.org/10.1002/14651858.cd011469.pub2.
37. Huang CH, Fu Y, Chi CC. Health-related quality of life, depression, and self-esteem in patients with androgenetic alopecia. JAMA Dermatol [Internet]. 2021 Jul 7;157(8):963. Available from: https://doi.org/10.1001/jamadermatol.2021.2196.
38. Khanna D, Khadka J, Mpundu-Kaambwa C, Lay K, Russo R, Ratcliffe J; Quality of Life in Kids: Key Evidence to Strengthen Decisions in Australia (QUOKKA) Project Team. Are we agreed? Self- versus proxy-reporting of paediatric health-related quality of life using generic preference-based measures: a systematic review and meta-analysis. Pharmacoeconomics [Internet]. 2022 Nov;40(11):1043-67. Available from: https://doi.org/10.1007/s40273-022-01177-z.
39. Zhou D, Zhang Z, Dou X, Xia F, Li X. Advances in the assessment of cosmetic outcomes, sensory alteration in surgical areas, and health-related quality of life of endoscopic thyroidectomy. World J Surg Oncol [Internet]. 2024 Feb 13;22(1):52. Available from: https://doi.org/10.1186/s12957-024-03307-7.
40. Wang H, Wang L, He Y, Yu G. Rotigotine transdermal patch for the treatment of neuropsychiatric symptoms in Parkinson's disease: a meta-analysis of randomized placebo-controlled trials. J Neurol Sci. 2018;393:31-8. Available from: https://doi.org/10.1016/j.jns.2018.08.003.
41. Khadka J, Kwon J, Petrou S, Lancsar E, Ratcliffe J. Mind the (inter-rater) gap. An investigation of self-reported versus proxy-reported assessments in the derivation of childhood utility values for economic evaluation: a systematic review. Soc Sci Med [Internet]. 2019 Sep 17;240:112543. Available from: https://doi.org/10.1016/j.socscimed.2019.112543.
42. Sun N, Zhang N, Lin LL, Wang LQ, Fan H, Yang JW, et al. Effect of acupuncture on emotional disorder in patients with chronic pain: a meta-analysis. Zhongguo Zhen Jiu [Internet]. 2020 Jun 12;40(6):657-63. Available from: https://pubmed.ncbi.nlm.nih.gov/32538020.
