# Supplementary material for: Towards recommendations for arts and health evaluation: a two-part review of Quality of Life scales used in mental health and arts research
Source: Front Public Health. 2026 Oct 12;14:1933623. doi: 10.3389/fpubh.2026.1933623 (PMC13700918; doi:10.3389/fpubh.2026.1933623)
Supplement: Supplementary file 3 [file Supplementary_file_3.DOCX]

**Supplementary File S3:** List of all Quality of Life Scales from all Reviews

Total number of Scales :71

|  | Scale | Frequency |
| --- | --- | --- |
| 1 | Euro-QoL(EQ-5D-3L/5L/Y) | 17 |
| 2 | SF-36 | 10 times |
| 3 | WHOQOL-BREF | 5 times |
| 4 | SF-12 | 5 times |
| 5 | Chronic Respiratory Questionnaire (CRQ) | 4 times |
| 6 | Disease-Specific Saint George’s Respiratory Questionnaire (SGRQ) | 3 times |
| 7 | Pediatric Quality of Life Inventory (PedsQL) | 4 times |
| 8 | European Organization for Research and Treatment of Cancer Quality of Life Questionnaire-C30 (EORTC QLQ-C30 global quality of life) | 3 times |
| 9 | Seventeen dimensional Health-Related Quality Of Life Measure (17D or HRQoL_17D) | 3 times |
| 10 | IBS-QOL | 2 times |
| 11 | QOLI: Quality of Life Inventory | 2 times |
| 12 | Quality of Life in Alzheimer’s Disease (QOL-AD) | 2 times |
| 13 | Clinical COPD Questionnaire (CCQ) | 2 times |
| 14 | Assessment of Quality of Life 6D (AQoL-6D) | 2 times |
| 15 | Child Health Utility 9D (CHU9D) | 2 times |
| 16 | Adolescent Health Utility Measure (AHUM) | 2 times |
| 17 | CHIP-CE = Child Health and Illness Profile–Child Edition | 2 times |
| 18 | Manchester Short Assessment of Quality of Life (MANSA) | 2 times |
| 19 | Quality of Life Enjoyment and Satisfaction Questionnaire (Q-LES-Q) | 2 times |
| 20 | Stroke Impact Scale (SIS) | 1 time |
| 21 | WHOQOL | 1 time |
| 22 | Fifteen Dimensional Health-Related Quality Of Life Measure (15D or HRQoL_15D) | 1 time |
| 23 | Sixteen-dimensional Health-Related Quality Of Life Measure (16D or HRQoL_16D) | 1 time |
| 24 | SF-6D | 1 time |
| 25 | Quality of Life in Late-stage Dementia (QUALID) | 1 time |
| 26 | World Health Organization Quality of Life - older adults module (WHO-QOL-OLD) | 1 time |
| 27 | Quality of Life Scale | 1 time |
| 28 | (EORTC Quality of Life Colorectal Cancer) EORTC QLQ-CR29 | 1 time |
| 29 | (EORTC Quality of Life Colorectal Cancer.)EORTC QLQ-CR38 | 1 time |
| 30 | Individualised Neuromuscular QoL Scale (INQoL) | 1 time |
| 31 | 39-item  (Parkinson's Disease Questionnaire) PDQ-39 | 1 time |
| 32 | 8-disease Parkinson's disease Questionnaire (PDQ-8) | 1 time |
| 33 | Stroke Specific QoL Scale (SSQoL) | 1 time |
| 34 | SF-8 | 1 time |
| 35 | Functional Assessment of Cancer Therapy (FACT-G) | 1 time |
| 36 | Person with Dementia Quality of Life (PWD-QoL) | 1 time |
| 37 | IBD-QOL | 1 time |
| 38 | Dutch RAND-36 HRQoL Survey | 1 time |
| 39 | MSQ, Migraine-Specific Quality of Life Questionnaire | 1 time |
| 40 | Asthma Quality of Life Questionnaire (AQLQ) | 1 time |
| 41 | AAQoL = Adult ADHD Quality-of-Life Scale | 1 time |
| 42 | AIM-A = The ADHD Impact Module–Adult | 1 time |
| 43 | AIM-C = The ADHD Impact Module–Child | 1 time |
| 44 | CHQ = Child Health Questionnaire | 1 time |
| 45 | KINDL-R = Instrument zur erfassung der gesundheitsbezogenen Lebensqualität von Kindern und jugendlichen | 1 time |
| 46 | Q-LES-Q-SF = Quality-of-life Enjoyment and Satisfaction Questionnaire Short Form | 1 time |
| 47 | YQOL-R = Youth Quality of Life–Research Version | 1 time |
| 48 | Gilles de laTourette syndrome-quality of life (GTS-QOL) scale | 1 time |
| 49 | Schizophrenia Quality of Life Questionnaire (SQLS) | 1 time |
| 50 | Thyroid surgery-specific questionnaire | 1 time |
| 51 | Thyroid cancer-specific quality of life questionnaire (THYCA-QOL) | 1 time |
| 52 | General Quality of Life Inventory (GQOLI-74) | 1 time |
| 53 | RAND-36 | 1 time |
| 54 | Pediatric Quality of Life Enjoyment and Satisfaction Questionnaire (PQ-LES-Q) | 1 time |
| 55 | Audit of Diabetes Dependent Quality of Life (ADDQoL) | 1 time |
| 56 | Diabetes Quality of Life (DQOL) | 1 time |
| 57 | Dermatology Life Quality Index (DLQI) | 1 time |
| 58 | Women’s Androgenetic Alopecia Quality of Life index | 1 time |
| 59 | Dermatology Quality of Life instrument in Turkish | 1 time |
| 60 | Modified Women’s Androgenetic Alopecia Quality of Life index | 1 time |
| 61 | Quality of Life Questionnaire/Interview / Making Decisions Empowerment Scale | 1 time |
| 62 | Lehman's Quality of Life Interview (QOLI)[AL6] | 1 time |
| 63 | Health Utilities Index 2(HUI2) | 1 time |
| 64 | Health Utilities Index 3(HUI3) | 1 time |
| 65 | Impact of Weight on Quality of Life -Lite (IWQOL-Lite) | 1 time |
| 66 | Eating Disorder Quality of Life (EDQOL) | 1 time |
| 67 | Eating Disorder Quality of Life Scale (EDQLS) | 1 time |
| 68 | Health Related Quality of Life in Eating Disorders (HERQoLED) | 1 time |
| 69 | Quality of Life for Eating Disorders (QOL-ED) | 1 time |
| 70 | Health-related Quality of Life (HRQoL-4) | 1 time |
| 71 | Pediatric Quality of Life Scale 4.0 Short Form 15 (PedsQL4.0 SF-15) | 1 time |
